# Development of an online suicide prevention program involving people with lived experience: ideas and challenges

**DOI:** 10.1186/s40900-021-00307-9

**Published:** 2021-09-08

**Authors:** Mareike Dreier, Johanna Baumgardt, Thomas Bock, Martin Härter, The 8 Lives Team, Sarah Liebherz

**Affiliations:** 1 Department of Medical Psychology, Center for Psychosocial Medicine, University Medical Center Hamburg-Eppendorf, Hamburg, Germany; 2 Department of Psychiatry and Psychotherapy, Center for Psychosocial Medicine, University Medical Center Hamburg-Eppendorf, Hamburg, Germany; 3 Department of Psychiatry, Psychotherapy and Psychosomatic Medicine, Vivantes Hospital Am Urban and Vivantes Hospital Im Friedrichshain, Charité-Universitätsmedizin Berlin, Berlin, Germany; 4 Association Irre menschlich Hamburg e.V., Hamburg, Germany

**Keywords:** Suicidality, Suicide stigma, e-mental health, Lived experience, Suicide prevention

## Abstract

**Background:**

Fear of stigmatization, self-stigmatization, and insufficient information can lead to secrecy, reduced help-seeking, lower self-esteem, and lower self-efficacy among people affected by suicidality or suicide. Therefore, we developed an online suicide prevention program aiming to improve knowledge about suicidality and suicide stigma.

**Methods:**

Inspired by the Australian program *The Ripple Effect*, a German team comprising people with lived experience of suicide, researchers, and clinicians was established for developing an online suicide prevention program. Therefore, we oriented on guidelines for evidence-based health information, for reporting on suicide, and on dealing with suicidality. The lived experience team discussed and developed concept, structure, and content of the program. This manuscript presents summaries of protocols from 16 team meetings and 3 written text reviews to outline the program development process. A summative evaluation 3 years after program development began was qualitatively analyzed based on thematic analysis.

**Results:**

Between 2018 und 2021, the lived experience team (n = 10) discussed possibilities of support in suicidal crises, attitudes towards suicide, content, and design of the online program. In a structured process, six members of the lived experience team reviewed the content. Eight persons shared their lived experience of suicide in video reports by focusing on constructive ways of dealing with suicidality or a loss by suicide, conveying hope and encouraging people to continue living. Team members recommended greater public and patient involvement from the application stage, as well as more financial and personnel resources.

**Conclusions:**

Through contributions to discussions and text reviews, the lived experience team shaped decisions in the program development process. While involving persons with lived experiences of suicide, it is important to consider that suicidality is 1. emotionally challenging, 2. a stigmatized issue, and 3. that the aspect of safety must be a priority. A distinction must be made between the duty of care based on actual risk and inappropriate overprotection. Hereby, transparency, autonomy, and a clear structure appeared to be helpful. For further research, we recommend a structured formative review process of the development of the program. Additionally, we recommend discussing the purpose and the specific design of the evaluation with a lived experience team in advance.

*Trial registration* German Clinical Trial RegisterDRKS00015071 on August 6, 2018.

**Supplementary Information:**

The online version contains supplementary material available at 10.1186/s40900-021-00307-9.

## Plain English summary

A team of persons with a lived experience of suicide and researchers jointly developed an online suicide prevention program. This online program addresses the taboo and stigmatized topic of suicidality from the perspective of people with lived experience. In our team are people who tried to take their own lives, thought about suicide, and people who lost a close person by suicide. For the program we wrote texts on suicidality and suicide on a scientific basis. In videos the team shared their experiences with suicide. The team told what helped them to continue living and gave them hope. Online program participants can read these texts and watch these videos. Participants can learn about suicidality and are encouraged to seek help if needed.

During the development of the program, the researchers created a working atmosphere characterized by respect, empathy, transparency, and openness. Dealing with suicidality can be emotionally stressful. The team felt comfortable and safe. The team was proud of the developed online program.

We describe strengths and weaknesses of the program development. We did not evaluate the involvement during the program development. If one wants to do this, the purpose should be explained to the team. The team should have a say in the design, such as what questions are asked.

Our program gained enormously from involvement of people with a lived experience of suicide. Involvement is possible and necessary even with complex and sensitive topics such as suicidality. Antistigma work should involve those affected. Involvement is important to create a credible program.

**Supplementary Information:**

The online version contains supplementary material available at 10.1186/s40900-021-00307-9.

## Background

Suicidality is a complex phenomenon that can be an expression and/or symptom of a mental illness, a consequence of a (mental or somatic) illness, or a consequence of stigmatization [1–3]. Most suicidal persons suffer from a mental illness [1, 4]. Effective psychological and pharmacological treatments for suicide prevention exist [5]; however, stigma, a lack of available and suitable support, a lack of perceived need, as well as insufficient information on mental health may limit help-seeking [6–11]. Suicide stigma not only affects persons who attempted suicide or have suicidal thoughts (e.g. self-stigma/internalized stigma, anticipated stigma, experienced stigma) [3, 12], but also persons close to the person concerned (i.e. stigma by association or courtesy stigma). For close persons a distinction can be made between persons caring for a person affected by suicidality [13–15], and persons who lost a close person by suicide [16–18]. In the latter case, stigma is linked to reduced psychological and somatic functioning and grief difficulties [19].

The use of online interventions can help individuals to inform themselves about mental health despite the stigma and taboo of a topic, to find local treatment options, and to prepare for contact with health care professionals [20–22]. However, internet use can entail risks for people in crisis situations, e.g. forum posts or website content can be of poor quality, incorrect, non-transparent or unreliable [23]. In addition, there are “pro-suicide” websites that can be classified as dangerous (e.g., websites that discuss the pros and cons of suicide methods, discuss ways of accessing suicide means, or provide lists of arguments why suicide is reasonable) [24, 25]. For these reasons, the option of low-threshold access to quality-checked websites that provide information on help available can also be particularly important for taboo subjects such as suicidality [26]. In addition to providing low-threshold information, research suggests that contact-based interventions can reduce mental health related stigma and increase help-seeking behavior [27]. Without the involvement of people affected, reducing suicide stigma or rather the taboo on talking about suicidality or a suicide seems hardly possible, as stigmatization can be reduced primarily through individual stories and personal encounters rather than plain texts [28].

### Aims of the online suicide prevention program “*8 Lives”*

The German e-mental-health portal www.psychenet.de [21, 29, 30] informs affected persons and relatives about mental disorders and treatment options, also involving people with a mental illness in the development process. The online suicide prevention program *8 Lives-Lived experience reports and facts on suicide* is accessible via https://8leben.psychenet.de. The program aims to contribute to this e-mental-health portal by

- (1) improving knowledge about suicidality by educating, raising awareness, and presenting professional help services,
- (2) reducing self-stigma and/or perceived suicide stigma of persons with a lived experience of suicide to enable them to open up to others about their condition and to seek help, and
- (3) increasing self-efficacy expectations of being able to cope with psychologically demanding situations.

The *8 Lives* program does not focus on symptom reduction, such as Internet-based cognitive behavioral interventions aimed at reducing suicidal ideation [31]. We classify our program in the area of suicide prevention through education and awareness. Although one chapter of the 8 Lives program includes some elements of cognitive behavioral therapy (CBT) that participants can optionally work on, the focus of the program is not a therapeutic one. The program does not aim to intervene at the symptom level, but to improve knowledge and reduce stigma.

The unguided online program *8 Lives* has been developed based on the Australian online program *The Ripple Effect* [32–34] which is aimed at rural farmers. *8 Lives* was developed by researchers and mental health experts in collaboration with persons affected by suicidality as well as relatives of affected persons. The content of the program was translated into German, broadened to a general population (e.g., the tailoring to the specific group of male farmers was removed), and adapted to the German cultural context. From our perspective, by including people with a lived experience from the same cultural context as the program's target group, it is possible to design the program in a culturally sensitive way and to consider attitudes toward suicide in the cultural context. In addition, before the program *8 Lives* was developed, the project team conducted a representative population survey in Germany to determine attitudes and knowledge about suicidality [35, 36]. Furthermore, the project group conducted a workshop with the creators of *The Ripple Effect* to obtain firsthand background information and experiences made. The rough outline of *The Ripple Effect* was adopted, while there were content and conceptual changes in *8 Lives.*

To our knowledge, there are no other online programs involving people with a lived experience of suicide that aim to reduce suicide stigma and increase knowledge on an individual level for five different target groups (1: suicidal thoughts, 2: suicide attempt, 3: loss by suicide, 4: concern for a close suicidal person, and 5: generally interested people).

### Aim of involvement of people with lived experience in research

Since people should have influence on research that affects them, the involvement of patients and the public (PPI) has become a prerequisite for applied health research [37]. This allows those affected to contribute their perspective, and share what matters to them, thereby improving quality and relevance of the research. Persons who are stigmatized or have internalized stigma can be reached more easily by involving those affected by suicidality [37–39]. This was especially crucial in our project with people with lived experience, as one of our aims was to reduce self-stigma and/or perceived suicide stigma of program participants through indirect video-based contact (i.e. video depictions). Encouraging people to talk about their own suicidality or the loss of a close person by suicide (which in many cases means breaking a taboo) and to seek help may be especially credible if other people who have a lived experience do the same.

In suicide stigma research, a community-based participatory research approach has been adopted to identify stereotypes, prejudices, and discriminations against people who attempt or die by suicide [12] and to define a factor structure for public suicide stigma [40]. In these projects, the *Stigma of Suicide Research Team* includes people with lived experience, suicide prevention advocates, mental health providers, loss survivor therapists, and researchers.

Involving mental health service users can add substantial value in the development of mobile applications for mental health [41], e.g. by providing feedback on required apps and key features before an app is developed [42], or e.g. by providing complementary elements to apps at an early stage of development [43].

Little is known about involving people with a lived experience of suicide in clinical research. This may be attributed to the fact that suicidality is an extremely sensitive topic and is therefore generally often defined as a study exclusion criterion (for both persons involved in research and for participants), and that suicidality as a complex phenomenon can be defined less clearly on the basis of a categorial diagnosis such as a mental illness [44]. Littlewood and colleagues [45] recently published a study protocol on evaluating the impact of patient and carer involvement in suicide and self‐harm research where they also reported on two studies involving persons with a lived experience of suicide: 1. ex-prisoners for research on suicide prevention in prisons [46] and 2. men attending hospital emergency department after self-harm [47].

The aim of this publication is to share our insights into the involvement of people with a lived experience of suicide in the development of an online suicide prevention program. Following the Guidance for Reporting Involvement of Patients and the Public (GRIPP2-SF-tool, see Additional File 1) [48], we describe outcomes of team meeting discussions and their influence on the program development, as well as ideas and challenges that emerged during the development of the program.

## Methods

The online program *8 Lives-Lived experience reports and facts on suicide* [German: *8 Leben-Erfahrungsberichte und Wissenswertes zum Thema Suizid*] was developed at the University Medical Center Hamburg-Eppendorf between 2017 and 2019. The study design consisting of a sequential explanatory mixed methods design with three measurement points (pre, post, follow-up) is described elsewhere [49]. Qualitative and quantitative data collection has been completed in September 2020; data analysis is currently ongoing. The online program is targeted at persons 18 years or older who are themselves affected by suicidality or are close to those affected. Furthermore, the program is also open to people who deal with suicidality in other ways, e.g., professionally, and to people interested in the topic in general. In this publication, we focus on the program development involving people with a lived experience of suicide.

### Project team

The project team comprised expertise from different backgrounds: 1. scientists specialized in medicine, psychology, and social science, 2. clinicians working as psychologists, psychotherapists, or physicians, and 3. persons with a lived experience of suicide (“lived experience team”). The lived experience team was involved in all phases of the development of the online suicide prevention program except the grant application.

### Recruitment of the lived experience team

The lived experience team was established using a purposive sampling strategy. At first, the project idea was presented twice to members of the trialogical association *Irre menschlich Hamburg e.V.* [50, 51]. This association comprises people affected by mental disorders, relatives of people affected as well as professionals working in the mental health care system. *Irre menschlich* organizes information, encounters and prevention projects on all aspects of mental health. One of its goals is to promote greater tolerance in dealing with others and sensitivity towards oneself. Twelve persons from *Irre menschlich* were interested in participating in the project *8 Lives*. Criteria for lived experience team membership were.

(1) having a lived experience of suicide i.e., suicide attempt, suicidal thoughts, loss of a close person by suicide, or caring for a close person who is suicidal,

(2) being currently in a stable situation, i.e., unlikely to get into a crisis because of the project involvement, and

(3) being at least 18 years old.

Potential team members were given a participant information form and provided written informed consent to participate. Additionally, a licensed psychotherapist assessed the individual's stability as sufficient for project participation. Lived experience team members decided the extent of their participation and had the right to revoke their participation at any time. This also included recalling the provided video and text material.

### Types and scope of involvement

There were four options of participating in the program development that could be flexibly combined. The lived experience team members could be involved.

(1) in the development of concept, structure, content, and design of the program,

(2) in the review of the text material,

(3) in personal video or written experience reports, and

(4) in creating short “digital postcard messages” that can be read by online program participants.

Team members were reimbursed for their participation; 80€ per text package that was reviewed, 80€ per person for a written experience report, and 800€ per person for a video interview. One member of the lived experience team had a scientific background and was also part of the project team (JB). Her work comprised 10% of a full-time-position and was reimbursed according to regular wages of public service.

### Protocols of the lived experience team meetings

Discussions of the lived experience team meetings and their results were recorded by written protocols. These were drawn up after the meeting and were sent to the lived experience team by e-mail; team members could add content or make amendments to the protocol. Based on the protocols, we summarized discussion points (see Additional File 2) and sorted the topics by *aims, concept (rejected ideas and evaluation), content, language, design,* and *structure of the online program*.

### Reviewing of online program texts by the lived experience team

For developing the information texts on suicidality, we oriented on the program *The Ripple Effect* [32], guidelines for developing evidence-based health information [52], national guidelines on depression [53], and international treatment guidelines on suicidality [54], current systematic reviews, e.g. [55], and recommendations for reporting on suicide [56]. The lived experience team reviewed all text materials in a structured process before its online release. We summarized the results of the written text reviews (i.e., information from documents in change tracking mode and comments from the lived experience team) and edited the proposed texts accordingly.

### Lived experience team

After the initial meeting, two persons decided against further participation due to lack of time. The final lived experience team (n = 10) differed regarding gender (n_female_ = 6, n_male_ = 3, n_diverse_ = 1), age (range = 19–73 years), working status (n_students_ = 1, n_employed_ = 4, n_unemployed_ = 1, n_pre-retired_ = 2, n_retired_ = 2) and profession (e.g. teacher, physician, truck driver, secretary, dispatcher, without vocational training, social scientist). Most team members reported personal suicide thoughts, suicidal behavior, or suicide attempts (n = 7). Three members were close persons of affected people; one person was close to a person affected by suicidality, and two persons lost a parent through suicide. Four persons were affected by suicidality or suicide in more than one way of the above named. During the two-year development period of the program, one person of the lived experience team decided to be admitted to a psychiatric hospital because of suicidality. This person did not relate his/her crisis to the program development and stated in retrospect that in the long run his/her active involvement led to lower suicidality. Another person described depressed mood and lack of drive within a limited period but did not associate this with the program development either.

### Reflections on the development process: summative evaluation

A questionnaire with 16 open-ended questions on three topics (*1: evaluation of involvement: enabling and hindering factors, 2: lessons learned from program development, 3: opinions on the lack of evaluation during program development*) was sent by e-mail to all active members of the lived experience team 3 years after the start of program development, approximately one year after the program went online, to reflect on the development process of the online program. To give examples of summative evaluation questions we used on topic 1: “*From your point of view, what went well/badly in the collaboration within the 8 lives team for the development of the "8 Lives" program? What was helpful/less helpful/not helpful at all?”;* topic 2: *“What would you recommend to another group if they wanted to develop an online program on suicidality / breaking the taboo? What would you do differently if we started again with the development of "8 Lives"?”*, topic 3: *"What are your thoughts/opinions on formative evaluation during program development?; “From your point of view, what speaks for/against an evaluation during the development of the program?"* One of the original ten lived experience team members died of a somatic condition. Two team members did not participate in the project after completing their tasks due to time constraints. The written free text responses from the lived experience team (N = 7) were qualitatively analyzed based on thematic analysis by Clarke & Brown [57] and presented and discussed in a team meeting. Based on three questions (“What were helpful/hindering/challenging factors in working with the lived experience team to develop an online suicide prevention program?”) the researchers also collected their reflections on the involvement.

### Lived experience team meetings

Sixteen meetings of the lived experience team were held between March 2018 and March 2021 at the University Medical Center Hamburg-Eppendorf, Germany (see Table 1). The meetings for discussing and revising content lasted 2–3 h; meetings focusing on video creation lasted up to 9 h. The schedule of the team meetings arose dynamically in the development process of the program, and the dates for all meetings were set jointly (Table 2).

**Table 1:** Overview of “lived experience team meetings”

1	***Kick-off Meeting**** (03/2018)* Presentation of the concept of the program (basic project *The Ripple Effect*) and scope of the lived experience team: different ways to participate
2	***Discussion: Review text package 1**** (05/2018)* Links to professional external help offers, knowledge about suicide and suicidality
3	***Discussion: Review text package 2**** (06/2018)* Understanding suicide attempts, suicide stigma, misconceptions/"myths" about suicide
4	***Discussion: Review text package 3**** (07/2018)* Strategies for dealing with suicidality/prevention strategies, communication, setting goals
5	***Preparation of the video experience reports**** (07/2018)* Sharing one's lived experiences in the group, preparing questions for the interview, writing one's own narrative for preparation if necessary; deciding what to reveal in the video; concerns regarding the videos
6	***Filming day 1**** (09/2018)* Members of the lived experience team (n = 4) share their suicide experience in a video interview
7	***Filming day 2**** (09/2018)* Members of the lived experience team (n = 4) share their suicide experience in a video interview
8	***Follow-up meeting video experience reports**** (01/2019)* View and discuss edited video sequences; discussion of an evaluation instrument for recording self-efficacy expectations, which is to be used for program evaluation (think aloud method)
9	***Technical check and digital postcard messages**** (03/2019)* Task distribution of the online program elements to be tested (focus: user experience, embedding of video sequences); Collecting and discussing “digital postcard messages”
10	***Presentation of the technically implemented online program**** (08/2019)* Possibility to include last feedback
11	***Joint presentation of the online program at a seminar series**** ("Social Psychiatry in Motion") (10/2019)*
12	***Final meeting**** (02/2020)* Restaurant visit; reflection on program development; conclusion of the lived experience team meetings
13	***Discussion on the continuation 1**** (12/2020)* Based on the evaluation results, the lived experience team discussed whether, which and how elements of the online suicide prevention program should be continued
14	***Discussion on the continuation 2**** (12/2020)* Further discussion on details of continuation of the program
15	***Discussion on the continuation 3**** (01/2021)* Further discussion on details of continuation of the program; discussion on summative evaluation
16	***Discussion on the continuation 4**** (03/2021)* Further discussion on details of continuation of the program; discussion of the summative evaluation results
17	***Discussion on the continuation 5**** (planned for 04/2021)*

**Table 2:** Kind of lived experience of suicide shared in the video reports

Age group, gender	Kind of the lived experience of suicide
70 s, male	One-time suicide attempt after a traffic accident
60 s, female	One-time suicide attempt in early adulthood after a breakup
20 s, male	Suicidal thoughts in a depressive episode
40 s, female	Recurring suicidal thoughts
50 s, female	Recurring suicidal thoughts in crises
40 s, diverse	Chronic suicidality with suicide attempts
50 s, male	Loss of mother by suicide
30 s, female	Loss of father by suicide

At the beginning of each meeting, the project status was summarized, and discussion topics were collected. These arose, for example, from the general concept of the online program, the text reviews, or previous meetings. A psychologist and a social scientist (MD, JB) or a psychotherapist (SL) led the meetings. One permanent contact person (MD) was defined for any requests and coordination. At the end of each meeting, pending tasks like text reviews, preparation of digital postcard messages, or interview questions were distributed. Finally, an outlook for the next meeting was agreed upon. The lived experience team received a protocol of the meeting by e-mail. A few days before the next meeting, a reminder was sent out with the planned meeting content. The lived experience team members could contact the project team (MD, JB, SL, TB) by e-mail and telephone between group meetings. There was also the possibility to make personal appointments with a project team member (MD, JB), which were mainly used for the preparation of the videos. A formative evaluation of the meeting, e.g., a structured feedback questionnaire after each meeting, did not take place.

## Results

In the following, results from (1) the lived experience team discussions on the program development, (2) the text reviews, (3) video reports on lived experience of suicide, (4) the content of the program, and (5) the results of the summative evaluation three years after starting the project are presented. Please note, that only an excerpt of the data can be presented below. Therefore, we highly recommend reading the supplementary files (see Additional files A2–A6), which present the discussion topics that arose in the team meetings on program development (A2), the results of the program text review (A3), questions prepared for video experience reports (A4), the entire content of the online suicide prevention program (A5), and the reflection on the development process (A6) in detail.

### Development discussions and joint consensus

Several topics regarding the online program development were discussed during the lived experience team meetings (see Additional file 2). The development of the online program was finalized based on joint consensus. In the following, some examples of team discussions are given:

#### Aims

Key messages of the online program were defined for different target groups. One key message was that help is available. That led to a discussion whether help is available e.g. in view of waiting times for psychotherapy or is always helpful (e.g. negative experiences in inpatient psychiatric stays or outpatient psychotherapies). Despite these experiences, we agreed to promote in the online program that it is worthwhile to seek help.

#### Concept

The team decided to create shorter video sequences and show video sequences on various topics of all eight lived experience team members in all five variants of the program, with the purpose of approaching the topics from different angles. Participants of the online program who e.g. indicated in the beginning to have lost a close person by suicide can decide to watch videos of people affected by suicidal thoughts and vice versa. However, we decided to make several distinctions based on the experience with suicidality a person has stated—because we considered that some statements may be more or less appropriate/helpful for some target groups.

#### Rejected ideas for content

One suggestion during the lived experience team meetings was to address the general attitude towards dealing with death and dying in society in the program. In the joint consensus, we decided against this idea because it would have gone beyond the scope of the program.

#### Content

Shame or fear of shame can lead to withdrawal behavior of persons affected by suicidality. The lived experience team did not find it helpful to "push" to disclosure and decided to leave the decision to the individual (autonomy). Therefore, respect for non-disclosure resonated in texts and video messages while at the same time the importance of support in a suicidal crisis and help offers were described.

#### Language/content

The use of humor in the online program was controversially discussed. Some stated that humor helps in dealing with suicidality. In the online program texts, we have dispensed with the element of humor, since we do not know how the anonymous participants feel about these messages. We have agreed that the digital postcard messages may contain personal statements with black humor. Also, in the lived experience video reports humor as a strategy can be explained.

#### Structure

The wish of the lived experience team was that only people who are seriously interested in the topic should have access to the program. Also, for this reason we implemented a login with email address.

### Text reviews

Information texts on suicidality, help options, and stigmatization were created and discussed in a first draft (MD, SL). A total of three text packages (between 9 and 15 letter size pages) were sent to the lived experience team by e-mail or mail. Five to six persons reviewed each text regarding comprehensibility, complexity, brevity, conciseness, quality, and completeness. All written comments were sent to the coordinator (MD) by a set deadline. Furthermore, it was possible to make oral comments by phone. Feedback was discussed in the next lived experience team meeting with the whole group and incorporated in the program texts. Overall, texts were described as comprehensive and concise. Some parts had to be adapted for the different target groups. In addition, simpler wording was suggested and incorporated. A summary of the written feedback of the lived experience team during the text review is provided in Additional File 3. The team rechecked the revised information texts during the technical review of the program. The program texts were finally approved by the lived experience team as well as the project team.

### Video reports on lived experience of suicide

After the first team meeting, two persons decided against disclosing their lived experience of suicide in a video report. One person decided to write an anonymous experience report. The decision not to disclose the lived experience in a video was made because it would have indirectly revealed the experience of the close suicidal person. The other person decided not to speak publicly about his/ her experience. Eight persons had decided to disclose a part of their lived experience of suicide in a video report. During team meetings, they discussed the concept and formulated possible questions for the videos (see Additional File 4). Finally, each person compiled his or her own questions (e.g., “How can I stay with the decision for life?”, “What helps me to deal with suicidal thoughts?”, “In which life situations did I think about taking my own life?”) in their preferred order. At the beginning of each video, there was a short introduction (name, age, profession, kind of the lived suicide experience). Some persons decided to use a pseudonym throughout the online program.

The videos and text messages aim to encourage others by showing them how to cope, and that there are people who experience or have experienced something similar. Thus, the lived experience team members’ personal stories including the experienced feelings are told, but the focus should then be on how to deal with the situation and how to cope with it. As suicide methods or places should not be mentioned, any references made to either one were removed retrospectively.

We filmed eight people on two days (four people on one day) to have enough time for reflection before and after the videos. We agreed on shooting the experience report in one-take. The participants could then add or re-record an answer. There were no time limitations concerning the video length. The edited video sequences (each the answer to a question, length of the answer < 1 min–12 min) were counterchecked and approved by the lived experience team member.

### Content of the online suicide prevention program

The program *8 Lives – Lived experience reports and facts on suicide* contains eight chapters composed of video reports about lived experience of suicide, fact sheets on suicidality, exercises based on cognitive-behavioral models, and worksheets (see Additional File 5). A user account is required and information on suicidality and lived experience videos are tailored to the kind of the participant’s suicide experience self-reported at the beginning of the program. There are five different variants of the program for participants with (1) suicidal thoughts, (2) suicide attempt, (3) loss by suicide, (4) concern for a close suicidal person, and (5) generally interested people. The help section is always visible during the program providing external professional support services via online links and telephone numbers of national and regional services, crisis lines and locations of emergency mental health services.

#### Technical check and final approval of the online program

The lived experience team tested the final technical implementation of the online program *8 Lives* twice regarding user experience, technical difficulties or errors, content, design, and spelling mistakes. A structured feedback sheet was used for this purpose. Changes were incorporated where technically possible. A responsive design was implemented, allowing the browser-based program being accessed through various devices (e.g. computer, smartphone).

### Summative evaluation three years after starting the project

All seven members of the lived experience team who were still actively involved in the project completed the summative evaluation questionnaire. The summative evaluation took place in March 2021, three years after the initial team meeting. The entire results of the summative evaluation (*1: evaluation of involvement: enabling and hindering factors, 2: lessons learned from program development, 3: opinions on the lack of evaluation during program development*) are presented in additional file 6.

#### Evaluation of involvement—Enabling and hindering factors

The lived experience team members described the respectful contact with each other, empathy, acceptance, care and understanding for each other, openness, and transparency as helpful overarching factors. Overall, team members experienced the project as valuable, took pride in the project, and found the pluralism of experiences helpful. The team members described sufficient opportunities to contribute one's own concerns to the project as well as a capacity for consensus in the group. The possibility to communicate with each other outside of the team meetings was experienced as helpful. The team described the moderation and coordination of the group (MD, JB) as empathetic and caring. The possibility of contact with project members (MD, JB, TB & SL) in potential (suicidal) crises, beyond meetings on different communication channels (by e-mail, phone or in person) created a safety feeling among the team. The team described the continuous information about the team meetings and the status of the project as positive as well as the clear focus of the team meetings. The time between the team meetings was described as important, also because there was an opportunity to prepare and follow up on the different topics. The team members positively emphasized the varied possibilities of involvement, the extent of one's own involvement being flexible as well as the financial reimbursement.

As a result of the project, the team members described various personal changes, which were evaluated as positive: Dealing more intensively with the topic of suicidality and suicide through the project, becoming aware of one’s own self-efficacy and ways of dealing with suicidality, developing a greater understanding of the function which suicidality has for each and every one individually, and having grown through the project.

Some team members reported ruminating on issues after team meetings, as well as having unpleasant feelings or thoughts activated in team meetings. Team members described this in part as emotionally taxing, in the long run, more as an internal coming into motion. The team members described that the strain could be well absorbed by the space in the project and team meeting framework. Being involved in the project and team where not much explanation was needed, was especially helpful as well as one-on-one conversations with lived experience team members or project members (MD, JB). To process the feelings and thoughts, some team members could additionally use psychotherapy with a psychotherapist independent of the project.

The time required for the project was mentioned as a hindrance: One team member mentioned that the project took too long. Two other team members thought that there was sometimes too little time in the team meetings or that there were too few team meetings. Two team members would have liked to have additional meetings that were not project related. One team member described difficulties with the dual role of a project team member and sometimes felt excluded, rejected, powerless, and not taken seriously enough in team meetings. The program was offline for the time of the evaluation and revision of the program. All team members reported back that the continuation of the program was not discussed early enough, which led to frustration.

#### Lessons learned from program development

Lessons learned from the perspective of the lived experience team (N = 7) three years after starting the project are presented in detail in additional information 6.2 (Additional file 6). The team members would have preferred a stronger public and patient involvement already at the application stage. More financial and personnel resources should have been planned, e.g., also for the continuation of the program. Team members recommended scheduling additional team meetings without a project focus. Interface between the technical and design online implementation and the project team could have been improved, so that persons who graphically and technically implement the program understand its concerns better and there is less loss of information and time.

#### Opinions on the lack of evaluation during program development

Retrospectively, five of seven lived experience team members were in favor of formative evaluation, one was neutral, and one team member was against formative evaluation. Team members noted that there was always an opportunity to provide feedback during the development process. In the summative evaluation, the team emphasized different advantages of formative evaluation during program development, e.g., regular feedback could help to perceive own needs early enough, to recognize problems or difficulties faster and to take necessary countermeasures. In conducting a formative evaluation, the teamwork may have been improved as a more in-depth assessment of the team’s needs could have potentially had a positive impact on group cohesion. A possible formative evaluation was also described as a possible stimulus for self-reflection and as an appreciation of the teamwork. As a further possible advantage, the team stated that regulated feedback may have made it easier to express criticism (e.g. for shy people). A formative evaluation was also seen as a possible relief for the researchers moderating the group (MD, JB). As a possible disadvantage of a formal evaluation the time factor was mentioned. Moreover, some team member stated as a disadvantage for a formal evaluation the possible interruptions of the (normal) workflow. The team may have focused on teamwork instead of the issue of suicide/suicidality and program development (e.g., constant judging of teamwork could be annoying, being and working together could become artificial, and/or circling around teamwork). A reluctance to use questionnaires for evaluation, especially tick-box ones, was also described. Formative evaluation was further described as a kind of "pseudo" feedback that could have exerted pressure, as well as creating a feeling like a "guinea pig". In the post evaluation discussion of the summative evaluation results, it became clear that it would be important to first explain the purpose of a formative evaluation (e.g. improvement of the work process and/or research) and to decide together as a team for or against the evaluation. If the team had decided to implement a formative evaluation, the team would have liked to discuss and determine the concrete form of the evaluation together (e.g., which questions, how often, etc.). The majority of the team members spoke out in favor of an oral evaluation with jointly defined questions that should be formulated in an open manner. According to the team, the evaluation should have not taken place too frequently (not after each team meeting, but rather at the beginning, in between and at the end of the whole development process). The evaluation results should have then been discussed orally in the team.

#### Researchers’ reflections on involvement

Additional to the summative evaluation of the lived experience team, we compiled reflections from a researcher’s perspective (N = 3) on this in detail in Additional File 6.4.

The lived experience team and project team agreed that in case suicidality increases, the project team should be informed in addition to the person’s health care professional. We found it helpful to discuss the possibility of a deterioration of a team member’s condition at the first meeting. One person could not attend one lived experience team meeting due to an inpatient clinic stay (because of an increase in suicidality). We discussed with the person and the team how we should deal with the specific situation and decided for a team meeting without the person. Retrospectively, we should have discussed how to deal with a possible inpatient clinic stay of a team member at the beginning when the lived experience team was formed because non-attendance due to an inpatient stay has an impact on the group. We established team rules, e.g. not to send potentially ambiguous e-mails regarding suicidal ideations or behavior to the project team, as it will be treated like an emergency which happened one time during the program development.

From the researcher’s perspective, a good working atmosphere, trust due to the dual role of a scientist with a lived experience, good group cohesion, a clear structure, autonomy, transparency and continuous contact, accessibility of the project team, appreciation of opinions and ideas and shared consensus, giving feedback on text reviews and status updates, and being informed about possible risks of participation were helpful in working together with the lived experience team to develop an online suicide prevention program. It was very important to avoid peer pressure, e.g., on disclosure.

## Discussion

Ten people with a lived experience of suicide were involved in the development of an online suicide prevention program aiming to enhance knowledge and to reduce stigma. We aimed at bringing participants of the program indirectly into video-based contact with people affected by suicidality (either self-affected or affected as a close person). In *8 Lives*, the video reports of the lived experience team tell “ups and downs” regarding dealing with suicidality or suicide while focusing on hope and overcoming a crisis. The lived experience team members could have a role model function and be beneficial for online program participants since they show how to talk about suicidality or suicide and offer ways of dealing with it in a constructive way [58]. Since all team members have lived experiences of suicide, they can be perceived as authentic and credible. Program participants were also encouraged to reflect upon their own thoughts and feelings. Additionally, they could anonymously share their own potential experience of suicidality or suicide. Besides this, we aimed at enhancing evidence-based knowledge on suicidality and possible help opportunities by implementing information texts. Thereby, we intended to empower participants to find opportunities for themselves, e.g., to talk about the tabooed topic of suicidality or a loss by suicide with close persons or professionals. For interested persons without a suicide experience, the program aimed at raising awareness of suicidality and stigma.

Our project showed that involving people with lived experience in research projects is possible and enriching, even for complex and sensitive topics such as suicidality. For the developed program, video reports of eight different team members with lived experiences of suicide as well as one written experience report created different possibilities of identification for online program participants. In addition to the contribution in the video reports, the input of the lived experience team essentially shaped the decisions in the development of the program at various levels: from overarching conceptual decisions such as the selection and level of depth of topics or how to present videos to participants, to detailed decisions such as wording of sentences.

While international guidelines for public patient involvement exist [59], there is comparatively less research on this topic in Germany. One guide on patient involvement in clinical research was published recently [60]. Our project involved people with lived experience of suicide and thus touched the field of clinical research, but the focus of the project was antistigma work and developing an online program. To our knowledge, little is reported about the involvement of persons with lived experience of suicide in research projects [45] and also about peer specialists in suicide prevention in mental health care services [61]. From a clinical perspective, persons with a lived experience of suicide should be involved in all stages of treatment development [44]. The same applies to antistigma programs [50]; certainly, one strength of the presented online suicide prevention program is the close involvement of the lived experience team over a period of roughly three years. Our team consisted of persons with different kinds of lived experiences of suicide in different phases of life (e.g., regarding age).

### Reflections on involvement of the lived experience team: Strengths and limitations

In summary, a trustworthy, friendly working atmosphere was established both from the perspective of the lived experience team and from the perspective of the researchers. The lived experience team described working with each other as equals and always felt safe during program development—despite the emotionally taxing topic of suicidality.

#### Safety plans

One issue that should generally be considered when involving people with lived experience [59] is safety. This aspect is crucial in general in participatory contexts and must be given special attention in suicide research [45]. Not involving people in research due to fear of possible crises seems immoral [59] or in a certain sense discriminating (e.g. “A person that once was suicidal will always remain suicidal.”). From our practical experience, the intention to behave in a non-discriminatory manner must be distinguished from the assessment of an actual functional impairment. By considering possible crises, we tried to not invalidate team members’ own assessments of stability. Therefore, we did not develop an individual safety plan for each team member within the framework of the project. As some team members have assessed themselves as stable, some team members had individual safety plans with their outpatient psychotherapists independent of the project. We agreed jointly that team members could contact the researchers and clinicians (MD, JB, SL, TB) by phone, mail, or in person if their condition worsened. In addition to the verbal discussion on safety plans, the option to contact the project team was noted in the written study information but was not further specified for various situations. In retrospect, a standard procedure could have been established in advance for deterioration of symptoms, an inpatient clinic stay, or an ambiguous message of a team member. From an antistigma perspective, consideration could be given to setting up a safety plan for all team members, including researchers. However, it must be assessed in advance how suitable such an approach would be for the team involved—especially if team members already have individual safety plans.

#### Emotionally challenging

Different memories related to suicide and suicidality came up in the team meetings and the team members needed to talk about these memories. With the topic of suicidality, this can also be emotionally challenging for the researchers who moderate the team meetings. We found it important to give space to these memories and personal feedback and then also to find the way back so that the team could continue to work together on the program development.

#### Continuation of the online program

The lived experience team did not feel sufficiently involved in the discussion on the continuation of the online program. The entire team wanted the online program to be available for users for an unlimited period. This continuation was not covered in the grant. In hindsight, the researchers would have involved the lived experience team earlier in the discussion on continuation of the program. After identifying the misunderstanding between the researchers and the lived experience team (the researchers initially planned to evaluate more results and underestimated the team's desire to continue the program), we held several team meetings and benefited from the lived experience team's opinions for further development, e.g., on participants’ feedback, while gaining more understanding on the researcher team's side about importance of data evaluation. The project team is implementing the continuation without reimbursement of efforts or wages. If possible, we recommend that a possible continuation of a project is already considered when applying for funding (provided that the evaluation results are promising). We recommend separating program development and program evaluation in terms of personnel.

#### Personal changes and empowerment

Some lived experience team members described that they were proud of their involvement in the project. Moreover, personal changes took place because of the project, such as becoming aware of one’s own self-efficacy and ways of dealing with suicidality/suicide, and a better understanding of the function which suicidality serves for each and every one individually. The researchers initially had not been aware of how important, empowering, and destigmatizing the project could be for the lived experience team itself (since it focused on stigma reduction among program participants). Our hypothesis is that through co-creation and/or involvement in such a suicide prevention project, in addition to initiating reflective processes, internalized suicide stigma of team members might be reduced. Potentially, this could be accomplished by sharing the lived experience in a safe setting among peers with a focus on empowerment and recovery, as well as the shared aim of helping others affected by suicidality.

#### Composition of the lived experience team

The lived experience team consisted mainly of persons who have had suicide thoughts, have exhibited suicidal behavior, or have had suicide attempts. Persons who have lost a close person by suicide were less represented as well as the perspective of persons who are worried about a suicidal person. Although all perspectives were represented in our team by at least one member and some team members have been affected in more than one way, the diversity of experience portrayed in our program could have been further increased. Since the relationship to the person who died by suicide plays a role in processing a suicide, a possible expansion of our program would be to add experiences from people with a different relationship status to the deceased person (e.g. experiences of a person whose child, partner, sister, brother, friend, or work colleague died by suicide). The perspective of persons who are worried about a close suicidal person could only be represented to a limited extent. From our point of view, it could be reasonable to present both perspectives, i.e., of the person affected by suicidality as well as of the close person. Persons who have a different cultural background and may have a different view of suicidality were given little consideration in the program.

Through participating in the trialogical association *Irre menschlich Hamburg e.V.,* some team members had previous experience in antistigma work and knew each other in advance. The latter enabled good group cohesion, trust, and support to be established quite fast. On the other hand, people within the same association might have similar views on certain topics, which could limit perspectives. To reduce this potential bias, people independent of an association, from other associations, or people affected by the topic for the first time could be included in further projects. Since the program also addresses people who are generally interested in the topic, but are not specifically affected, it would have been reasonable to include them in the development as well.

#### Lack of formative evaluation

The entire project focused on the evaluation of the online program in a pre-post design, not on evaluating the participatory development process. The evaluation of program development could have been more standardized, planned further in advance, and more operationalized [62–64]. Distortions may occur as we have compiled the discussion points retrospectively solely based on protocols and written text review comments. For instance, a regular written quantitative evaluation of the lived experience team meetings directly after a meeting, both for people with a lived experience and the researchers or audio recordings of the meetings, may have improved the methodological quality (e.g. no distortion by memories, better objectivity, and replicability of findings). One outcome of the summative evaluation three years after starting the team meetings was that the lived experience team felt there were sufficient opportunities for feedback. Nevertheless, from the perspective of most of the team members, a formative evaluation should have been conducted. When considering a formative evaluation, the team made clear that it would have been necessary to explain the purpose of such an evaluation. At the beginning of the development phase, a transparent discussion about the advantages and disadvantages of a formative evaluation would have been necessary. The team would have wanted to decide for themselves whether a formative evaluation should take place or not and for what purpose (quality improvement of work, research, etc.). The team would have wanted to determine the specific questions of the evaluation, as well as the frequency and other aspects of the evaluation. We recommend this for similar projects—although the process takes time. It should be considered that additional evaluation or reflection can be demanding for the team. From a researcher's perspective, we would additionally recommend considering having a formative evaluation conducted for all team members (with and without lived experience) by independent researchers. If only the persons with a lived experience of suicide were interviewed about program development, it would create an imbalance and, in a certain sense, turn the persons back into ‘research objects’. Therefore, if deciding to use a qualitative approach to suicide stigma program development (e.g., analyzing audio recordings of team meetings), we would recommend considering the evaluation of statements of involved people without lived experience of suicide, such as statements of researchers.

## Conclusion

We described the development process of the online suicide prevention program *8 Lives* involving a lived experience team by summarizing topics discussed in the lived experience team meetings, showing results of the text review on information on suicidality, and describing the creation of lived experience of suicide video reports. As a result of a summative evaluation, we recommend a formative evaluation of the development process, of which the purpose and design is co-determined by the people involved. Involving people with lived experience of suicide is possible and enriching for research projects and can also be empowering for the people involved. From our perspective, for the development of antistigma programs the involvement of people with lived experience is essential to create a credible program.

## Supplementary Information

**Additional file 1.** GRIPP2-SF.

**Additional file 2.** Discussion of the lived experience team on program development.

**Additional file 3.** Results from text review.

**Additional file 4.** Questions for lived experience reports.

**Additional file 5.** Content of the online suicide prevention program.

**Additional file 6.** Reflection on the development process. **6.1** Evaluation of involvement: enabling and hindering factors in program development. **6.2** Lessons learned from program development. **6.3** Opinions on the lack of evaluation during program development. **6.4** Reflections on involving a lived suicide experience team in an online suicide prevention program. Development from researchers’ perspective.

## Data Availability

All relevant data are within the manuscript and its additional information files.
