# Supplementary material for: Development of an online suicide prevention program involving people with lived experience: ideas and challenges
Source: Res Involv Engagem. 2021 Sep 8;7:60. doi: 10.1186/s40900-021-00307-9 (PMC8424946; doi:10.1186/s40900-021-00307-9)
Supplement: Supplementary file 1 — Additional file 1. GRIPP2-SF. [file 40900_2021_307_MOESM1_ESM.docx]

GRIPP2- Short Form

**Additional file 1.** **GRIPP2-SF**

| **Section and topic** | **Item** | **Reported on page No** |
| --- | --- | --- |
| 1: Aim | To develop an online suicide prevention program that aims to improve knowledge about suicidality, including help opportunities and to reduce stigmatization of suicidal persons as well as stigmatization of those who lost a person by suicide. People with a lived experience of suicide should be involved in all stages of development of this online program. | 4-5 |
| 2: Methods | For developing an online suicide prevention program, a lived experience team consisting of ten people was founded. Most team members reported personal suicide thoughts, suicidal behavior, or suicide attempts (n=7). One person was close to a person affected by suicidality. Two persons lost a parent by suicide. By the time, the program was launched online, there were twelve meetings of the lived experience of suicide team along with scientists and clinicians who coordinated the project. Between meetings, team members worked on various tasks to develop the program. The lived experience team members were involved in (1) the development of the concept, structure, content, and design of the program, (2) the review of the online program’s text material, (3) personal video or written experience reports, and (4) creating short “digital postcard messages” on suicidality that can be read by online program participants.  Three years after the project started, the active lived experience members (N=7) answered a summative evaluation on the program development process. The lived experience team contributed to edits of the paper and are coauthors. | 6-9 |
| 3: Study results | The lived experience team contributed to the study in several ways, including:  -Identifying relevant aims for the online suicide prevention program, as well as working on the program concept and structure, and relevant content from an expert by experience perspective.  -Reviewing texts for the online program.  -Sharing a lived experience of suicide in video reports for online program participants.  -Contributing to edits of the paper.  In retrospect, the lived experience team described working with each other as equals and always felt safe during program development, despite the emotionally taxing topic of suicidality. The team members would have preferred a stronger public and patient involvement already at the application stage. More financial and personnel resources should have been planned, e.g., also for the continuation of the program after the research project is completed. | 10-15 |
| 4: Discussion and conclusions | Our project showed that involving people with lived experience in research projects is possible and enriching, also for complex and sensitive topics such as suicidality. Through contributions to discussions and text reviews, the lived experience of suicide team shaped decisions in the program development process. When involving persons with lived experience of suicide, it is important to consider that suicidality is 1. emotionally challenging, 2. a stigmatized issue, and 3. that the aspect of safety must be a priority. A distinction must be made between the duty of care based on actual risk and inappropriate overprotection. For the development of antistigma programs, the involvement of people with lived experience is essential to create a credible program that is relevant to others. | 16, 20 |
| 5: Reflections/critical perspective | The program concept was already roughly defined by the grant proposal. The process of developing the grant proposal did not involve people with a lived experience. The possibility of a continuation of the program should be discussed early. We recommend a formative evaluation of the development process, of which the purpose and design is co-determined by the people with lived experience involved. | 17-20 |
