# Supplementary material for: Development of an online suicide prevention program involving people with lived experience: ideas and challenges
Source: Res Involv Engagem. 2021 Sep 8;7:60. doi: 10.1186/s40900-021-00307-9 (PMC8424946; doi:10.1186/s40900-021-00307-9)
Supplement: Supplementary file 2 — Additional file 2. Discussion of the lived experience team on program development. [file 40900_2021_307_MOESM2_ESM.docx]

Discussion of the lived experience team on program development

Note: Table I presents opinions and ideas of the lived experience of suicide team on program development during team meetings. If there was no divergent discussion, only the joint consensus is presented in the table. The summarized discussion topics are based on team meeting protocols.

Additional file 2. Summary of discussion points of the lived experience team during the team meetings based on protocols.

|  | **Discussion of the lived experience team and/or joint consensus of the lived experience team and project team** | |
| --- | --- | --- |
| *Aims* | | |
| **Topic** | **Joint consensus** | |
| *Key messages of the online suicide prevention program* | Key messages to deliver to the participants:   - Talking about suicidality or a loss by suicide is possible. - Help is available. - Being sensitive about suicidality and knowing how to get help – despite suicide stigma - might prevent a suicide. | |
| *Messages for different target groups* | *For those affected by suicidality*   - We have decided to live on and recommend doing so. - Dealing with suicidality in a constructive way is possible. - There is hope. (We try to convey hope for continuing living.)   *For those who lost a close person by suicide*   - We wish that they can express themselves and are able to exchange about the pain of loss and process the loss - which can take a lot of time.   *For those who worry about a close suicidal person*   - We wish that they enter an exchange with the suicidal person, encouraging the person to seek help, but also to recognize and acknowledge their own limits.   *For participants interested in the topic of suicidality:*   - Be sensitive and tolerant. - Know what to do if you or a close person might be in a suicidal crisis (talk about it to a close person and seek help) | |
| *General concept* | | |
| **Topic** | **Discussion topic in the lived experience team meeting** | **Joint consensus** |
| *No crisis intervention* | Some stated, that the online program would overburden people in an acute suicidal crisis or people with severe mental disorders or people in acute grief (e.g., texts and videos are too long). | It is important to clarify for participants that the program is no crisis intervention. The "find help" section will be always visible providing external support services via online links and telephone numbers of national and regional services, crisis lines and locations of emergency mental health services. |
| *Unguided program* | Close monitoring of the program is not possible. The participants move anonymously through the program. | It is important to emphasize that it is an unguided suicide prevention program aiming to increase knowledge (e.g., on help offers) and to reduce stigma to seek help. |
| *Different target groups* | The program aims to address different target groups, which leads to a balancing act of addressing different forms of stigma. Addressing those affected by suicidality themselves and persons close to those affected and persons interested in the topic is challenging in program development. | This implementation is planned in the pilot project. For further developments, it would be possible to concentrate even more on specific variants of the five variants of the program. Idea to encourage participants in the program to adopt different perspectives is supported by the team. |
| *Assignability to one target group* | Online program participants need to choose in the beginning what kind of lived experience of suicide concerns them. It is possible to be affected by suicidality/suicide in different ways. Possible to combine different program variants? | It is a good idea to address different types of affliction and create individual programs based on the information given at the beginning. However, there are many possible combinations. A more tailored implementation is not possible for us, neither in terms of content nor technically. The participants will be asked in the beginning which experience currently affects them most.  We have agreed that once a participant has completed the program, he or she has access to all videos, postcards, and worksheets. |
| *Video reports: Tailoring for online program participants?* | The idea inspired by *The Ripple Effect* was to create one or two longer videos (about 5-15 minutes) of each team member and tailored to specific target groups (by kind of suicide experience, age, gender). | We decided to 1. create shorter video sequences and 2. show video sequences on various topics of all eight lived experience team members in all five variants of the program, so that the topics are approached from different angles. Reasons: Shorter sequences are more responsive and less overloaded for participants; to achieve more variety and diversity; more characteristics than kind of suicide experience, age and gender determine whether what one person shares in the video is helpful for another person.  Participants of the online program who e.g. indicated to have lost a close person will also see videos of people affected by suicidal thoughts and vice versa.  However, the team decided to make several distinctions based on the experience a person has had with suicidality - because some statements were more or less appropriate/helpful for some target groups; e.g. for participants who have lost a close person by suicide the program focuses on grief. |
| *Transdiagnostic* | A positive aspect is that the program is not intended to refer to individual diagnoses but to suicidality and suicide. | |
| *Title of the online program* | Should convey hope and be easy to understand what it will be about; e.g. word "prevention" (in German: “Prävention”) is too difficult | |
| *Inspiration for self-reflection* | Unlike a documentation, the participants should not be mere recipients of the videos. | We give the participants the opportunity to self-reflect within the online program. |
| *Depth of topics* | Dealing more deeply with e.g., the issue of suicide stigma? | Experienced as sufficient. Too complex in a non-scientific context. |
| Suicide stigma may have a suicide preventive effect | The program’s aim to reduce stigma could be misunderstood because certain aspects of suicide stigma may also have a suicide preventive effect. [1,2] | From the team’s perspective, it is important in the suicide prevention program not to “normalize” or “accept” suicides. The program targets talking about suicidality to get help. In our program, we clearly advocate seeking help and continuing to live. |
| *Risk of reproducing stigma* | During the development of the program, the translation of questionnaires, and the text development for suicide stigma, the idea came up that by focusing on stigma experiences, stigma is reproduced.  On the one hand, we need to assess and address stigma; on the other hand, we give participants ideas about stigma that they might not experience in their environment. | We focused on what is experienced as helpful for those affected and their relatives/close persons and to focus on constructive models for dealing with suicidality instead of focusing on what could be stigmatizing. |
| *Rejected ideas for content and program features* | | |
| **Topic** | **Discussion topic in the lived experience team meeting** | **Joint consensus** |
| *Explaining more about prominent persons who died by suicide* | In the sense of the message that, for example, depression and suicides occur in all social classes, one person suggested that the program could report on celebrities who have died by suicide. | We decided against it, among other things because it is quickly sensationalizing, could increase glorification and romanticizing of suicides. Identification with a prominent person who has suicided could lead to imitation suicides. |
| *Interviewing train drivers* | One person suggested that people such as train drivers who have experienced how a person has suicided could be interviewed for the program. This could be used to discourage many people from suicide if the drivers’ emotional strain is shown. | We decided against this idea because it was not in the scope of the program. Moreover, we do not report on suicide methods. Inducing guilt (fear, shame) may prevent suicide for some persons affected by suicidality, for some it will not. It could change suicide methods but not suicide itself. |
| *Dealing with death and dying in society* | One suggestion was to address the general attitude towards dealing with death and dying in society in the program. | We decided against this topic because it would go beyond the scope of the program. |
| *Right to suicide* | Some remarked that they believe that in principle people have a right to suicide, in the sense of the autonomy of each person and the free will of the human being. | We decided not to present philosophical considerations on this in the program - as this would go too far. We agreed that in crises situations the view of the world is often limited. Then a "right to suicide" (which may help some people as a concept) could be taken up wrongly by others. We decided not to address this issue in an online program. |
| *Assisted suicide* | One person suggested that the program could take up the debate in Germany on assisted suicide in seriously ill patients. | We decided that the issue of assisted suicide would go beyond the focus of the program and is therefore not addressed. |
| *Addiction and suicide* | Idea for possible topic: "Slowly emerging suicide", especially in the field of addiction | Beyond the scope of the program (although e.g. alcohol addiction was discussed as a risk factor) |
| *Chat/Forum* | Integrate a chat or forum where program participants can exchange experiences with each other. | For program participants, there will be no possibility to get in contact with other participants (e.g. via chat, forum), because the amount of support would be too large and cannot be accomplished within the project. A digital postcard message can be sent anonymously. |
| *Evaluation* | | |
| **Topic** | **Discussion topic in the lived experience team meeting** | **Joint consensus** |
| *Program evaluation* | There are far too many questions in the beginning and end for evaluating the program. It is hard to imagine that participants are willing to answer all these questions. | We offered a questionnaire as a table instead of single items per page.  We explained why there need to be questions – also the same questions at the program end. |
| *Suicidal stigma not separable for an affected person* | Stigma of suicide scale- self version: Suicide stigma may overlap with other stigmatization experiences; this cannot be clearly separated for a person affected. | This is a very good idea. Unfortunately, we cannot answer this question within our evaluation. It is assumed that the constructs of suicide stigma and mental illness stigma, while similar, are different. It is theoretically conceivable that other experiences also lead to internalized stigma. To find out, we would need more assessment tools. |
| *Questionnaire* | Measuring “empowerment”/ Self-efficacy expectations of dealing with psychologically difficult situations; a newly developed questionnaire with 7 items was tested and discussed. [Not presented here in detail.] | |
| *Content* | | |
| **Topic** | **Discussion topic in the lived experience team meeting** | **Joint consensus** |
| *Suicidality as a continuum* | Suicidal thoughts as human thoughts: Almost everyone has had suicidal thoughts (even if only as an undefined theoretical possibility).  Important: Distinction between wanting to die or needing a break from life. | We decided to present suicidal thoughts as a continuum. [3,4] |
| *Help availability* | There was a critical discussion on whether help is available (e.g., waiting times for psychotherapy, unavailability of the telephone crisis service) or is always helpful (e.g., negative experiences in inpatient psychiatric stays or outpatient psychotherapies). | Despite these experiences, we agreed to promote that it is worthwhile to seek help and not to give up if a person does not find the help, he/she is looking for. |
| *Do not embellish help* | Some members of the working group described negative experiences with the health care system. Help should not be embellished, as this would be inauthentic. On the other hand, psychiatry should not be demonized, it can be a safe place. | Our message is that if it does not work with one person / health care professional, it is important to try again - if necessary, with another person. |
| *Principle option to suicide* | Some report that the principal possibility of suicide helps or has helped them to stay alive. See suicide as one of many options but try the many other options first. Having overcome crises can give strength for living on. | We decided that as a personal experience report we can address this issue in the program emphasizing the positive outcomes (deciding against suicide). |
| *Importance of time* | In a suicidal crisis it is important not to follow the impulse but to gain time and thus stay alive. | |
| *Safety plan* | Add a safety plan in case of suicidality. | Since the online program is no crises intervention, we decided to explain the concept of a safety plan and to indicate that the plan should be worked out with another person, preferably a psychotherapist or physician. We provide links to external quality-controlled safety plans. |
| *Disclosure of suicidality* | Possible fear, fear of shame, rejection, etc. among those affected can lead to withdrawal behavior. Reasons for this can be due to suicide stigma or mental illness stigma. | In the case of suicidal tendencies, however, it is particularly important – maybe even more important than with mental illness in general - to confide in others/professionals. |
|  | The program should not pretend that disclosure is not associated with risks. Therefore, identify the advantages and disadvantages of disclosure. If a participant in the online program has so far decided against disclosure, it would be invalidating not to give reasons against disclosure.  Some lived experience team members have not talked about the suicide attempt for a long time and this helped them in dealing with it. | We do not find it helpful to "push" to disclosure; it is important to leave the decision to the individual (autonomy). Respect for non-disclosure should resonate in texts. Can also be addressed in video experience reports. |
|  | Depends on the time: In case of acute suicidality it is important to confide in others (/professionals). | We clearly stated to participants of the program who have indicated suicidal thoughts that professional help should be sought, especially in acute crisis. |
|  | Disclosure from the perspective of a person of a suicidal relative: Making it public would involuntarily out the person affected. Strong need to protect the family, the affected person, and one’s own person by remaining silent. Possible reasons: Fear of exclusion from the family. Fear of embarrassing family and excluding them from the community. | |
| *Revenge* | It is useless to take revenge on the offender by suicide. That would be a very high price. The best revenge is to stay alive. | We only addressed this issue by a digital postcard message because it would go beyond the scope of the program. |
| *Talking on guilt in video experience reports* | If possible, do not blame anyone in the video; however, feelings of guilt can be addressed. | |
| *Digital postcard messages* | We developed 59 messages. Collection of short sentences that are embedded in the program (“Digital postcard messages”) - examples of questions for postcards:  What helps you in difficult life situations? What would you wish for from others in dealing with suicidality? What can you share with others that gives them courage?  Trying to make sure that the postcard messages do not become a “calendar motto imitation”. | |
| *Suicide numbers* | We decided against showing suicide numbers [prevalence rates of suicide] on the front page as they are not important for the individual experience. In the chapter on suicidality, however, the numbers are taken up. | |
| *Language* | | |
| **Topic** | **Joint consensus** | |
| *In general* | We want to show sympathy, compassion, understanding and help - just as it is possible in an online program without direct contact. | |
| *Texts* | The program texts are written in an objective, sober, clear, and informative tone.  In some parts of the program texts we address the persons directly, showing empathy and focus on conveying hope. | |
| *Videos* | The team members are free to design the videos. In contrast to the texts, the individual experience reports on suicidality or a loss by suicide are emotional. No suicide methods are mentioned, and the focus is on dealing constructively with suicidality. Metaphors can be used (e.g. suicidal thoughts as annoying buddies who come by but leave). | |
| *Humor and suicidality* | Humor, especially black humor, can help in dealing with suicidality. One person describes that humorous, even macabre statements about suicide or death, makes her/him smile a bit. The person describes that humor takes the weight and emotional tension and gives some lightness. Thus, humor can make suicidal thoughts become smaller. The thoughts are then not gone, but it is better to endure.  One idea was also to choose a humorous title for the online program. | In the online program texts, we have dispensed with the element of humor, since we do not know how the anonymous participants feel about these messages. Humor is something subjective, perhaps someone feels hurt by it.  We have agreed that the digital postcard messages may contain personal statements with black humor. Also, in the lived experience video reports humor as a strategy can be explained. |
| *Plain language* | The program could be translated into plain German language (or other languages) - so participants can choose between different versions. | Cannot be realized within the project for resource reasons. |
| *Subtitles for videos* | Add subtitles to the video sequences. | Cannot be realized within the project for resource reasons. |
| *Design* | | |
| **Topic** | **Discussion topic in the lived experience team meeting** | **Joint consensus** |
| *Showing one’s face?* | How to create the front page of the online program?  In some antistigma campaigns, people are not shown in a recognizable way because they prefer to remain anonymous.  Other campaigns use pre-produced images of people (stock photos) that are easily available or use actors to convey a message. We have discussed these options in several meetings and decided against them. The idea was, that it is difficult to convey that one should entrust suicidal thoughts and feelings to someone when we use photos of models or actors. | Seven team members have provided a photo for the frontpage with the option of revoking it at any time. Full names are not published.  For potential participants it is not clear from the outset who the persons in the photos are and whether they have a lived experience of suicide.  We decided for three statements below the photos:   - Every suicide or suicide attempt affects family, friends, and colleagues. - Almost everyone knows someone who died by suicide. - Many of us remain silent instead of talking about suicide and suicidality. |
| **Topic** | **Joint consensus** | |
| *Design of the online program* | For the whole program: It was important to choose a rather colorful, appealing design. Drawings to support the texts. | |
|  | For postcard messages: Pictures from nature, which express and underline strength, time, hope, encounter, and other supporting elements | |
|  | A web design agency was responsible for technical implementation and the design of the program. The design agency’s drafts were discussed in team meetings. Discussion is not presented here in detail. | |
| *Structure* | | |
| *Login* | The program is available behind a login. The login is seen as a small protection of the lived experience reports. The wish was that only people who are seriously interested in the topic should have access. Furthermore, the login creates an indicated prevention by self-assignment (and assignment to a variant of the program) instead of a universal access possibility. | |
| *Skipping between chapters* | Skipping between chapters is not possible - there were different opinions about this. We decided that after a participant has completed the post-assessment, it is possible to access all content chapters. | |
| *Structuring elements* | Decided on pause options after each chapter, progress bar, overview navigation page, one color per chapter. | |
| *Other* | | |
| *Allowance* | The team discussed that an expense allowance should also be paid for text reviews and experience reports (not only for personal video reports as proposed). With a view to the budget, it was jointly decided that no additional expense allowance could be paid for the meetings. | |

**References**

[1] Oexle N, Mayer L, Rusch N. [Suicide stigma and suicide prevention]. Nervenarzt. 2020;91(9):779-84.

[2]Chen JA, Courtwright A, Wu KC. The Role of Stigma and Denormalization in Suicide-Prevention Laws in East Asia: A Sociocultural, Historical, and Ethical Perspective. Harv Rev Psychiatry. 2017;25(5):229-40.

[3] Wolfersdorf M, Etzersdorfer E. Suizid und Suizidprävention. Stuttgart: Kohlhammer Verlag; 2011.

[4] Schomerus G, Angermeyer MC, Baumeister SE, Stolzenburg S, Link BG, Phelan JC. An online intervention using information on the mental health-mental illness continuum to reduce stigma. Eur Psychiatry. 2016;32:21-7.
