# Supplementary material for: Development of an online suicide prevention program involving people with lived experience: ideas and challenges
Source: Res Involv Engagem. 2021 Sep 8;7:60. doi: 10.1186/s40900-021-00307-9 (PMC8424946; doi:10.1186/s40900-021-00307-9)
Supplement: Supplementary file 3 — Additional file 3. Results from text review. [file 40900_2021_307_MOESM3_ESM.docx]

**Results from text review**

Additional file 3. Summary of the written feedback of the lived experience team during the text review.

| **Topic** | **Feedback and recommendations** |
| --- | --- |
| Text package 1 (N=6):  Links to external help offers, knowledge about suicide and suicidality (9 DIN A4 pages + checking external links) | |
| **Help availability** | Emphasize:   - ANY hospital emergency room can help in a suicidal crisis; go there and wait for help. - In an emergency also ring the neighbor’s doorbell or ask unknown persons for help. - In a suicidal condition it is very important not to be alone; seek conversations and closeness to others. |
|  | Rephrase "Inpatient psychiatric care" because "psychiatric" may discourage people; better just call it "inpatient care". |
| **Links to external help offers** | Collected links were helpful and comprehensive. |
|  | Note for people in acute crisis is suitable. |
|  | It may not be clear to relatives/close persons when they should call the emergency services/police, i.e. when a person is in an acute suicidal situation. Define “acute”. |
| **Suicide history** | Explain more about the historical view on suicide, as this is a source of suicide stigma. |
| **Terms for suicide** | It is important to address the distinction between the terms self-murder (German “Selbstmord”), free death/voluntary death (“Freitod”), self-killing (“Selbsttötung”) and suicide (“Suizid”). And to explain why we choose one term (suicide) in the program. |
| **References** | In order not to disturb the flow of reading, no references are given in the text. We have listed all references in the library sorted by topic. |
| Text package 2 (N=5):  Understanding suicide attempts, suicide stigma, taboo, misconceptions/"myths" about suicidality (15 DIN A4 pages) | |
| **Term “Stigma”** | Explain the origin of the word "stigma". |
| **Misconceptions about suicidality** | Add that suicidal thoughts can also disappear completely. |
| **Talk about suicide** | From the perspective of a family member who lost someone: If you open yourself up to this topic, it can be a cry for help. But opening up is also very relieving, regardless of how the environment reacts. 🡪 Discussed in the lived experience team that it does not have to be relieving for all relatives to open up. It also depends on the timing. |
| **Difference between received and perceived stigmatization** | Here, self-perception and external perception play a role. From the perspective of a family member who lost someone, one tends to mix it up and take some behavior personally. |
| Text package 3 (N=5):  Strategies for dealing with suicidality/prevention strategies, communication, setting goals (13 DIN A4 Pages) | |
| **Safety plan for suicidality** | Add a safety plan in case of suicidality. 🡪 Since the online program is no crises intervention, we decided to explain the concept of a safety plan and to indicate that the plan should be worked out with another person, preferably a psychotherapist or physician. |
| **Feelings** | Include "radical acceptance" as a technique? 🡪 Too complex to introduce within the scope of the program. We decide to sensitize for different feelings. |
| **One idea/input for every category** | Good idea to divide the strategies for dealing with suicidality/prevention into the four categories of behavior, thoughts, feelings and body. |
|  | Good initial input for those affected. Probably too little for those experienced in psychotherapy, but good repetition. |
|  | Note that it is up to you to decide what seems appropriate for you. 🡪 Possibility to skip coping strategies for online program participants. |
|  | More techniques would go beyond the scope of the program. |
| **Worksheets** | A worksheet for each strategy should be made available for download so that people can save it. |
| **Disclosure** | It is important that the advantages and disadvantages of disclosure are mentioned. Maybe add more disadvantages. |
| **Communication** | For relatives/close persons: If a person has suicidal thoughts, it is often all about talking, listening, accepting and to be there. It is important to take suicidal thoughts seriously and not to downplay them out of one’s own insecurity. |
| **Different target groups** | Stronger adaptation for the different target groups necessary. |
| All text packages | |
| **Overall feedback** | The texts are comprehensive, concise, interesting, and give a lot of input; well elaborated. |
|  | For people in a suicidal crisis or with a severe mental disorder they may be too long/too complex. |
|  | Texts are sufficiently complex. |
| **Wording** | In text passages simpler wording as well as words with other connotations were suggested and incorporated. These changes will not be presented here in detail. |
