# Supplementary material for: Development of an online suicide prevention program involving people with lived experience: ideas and challenges
Source: Res Involv Engagem. 2021 Sep 8;7:60. doi: 10.1186/s40900-021-00307-9 (PMC8424946; doi:10.1186/s40900-021-00307-9)
Supplement: Supplementary file 4 — Additional file 4. Questions for lived experience reports. [file 40900_2021_307_MOESM4_ESM.docx]

**Questions for lived experience reports**

Additional file 4. Questions used in the lived experience of suicide video reports.

| **Question for talking about a lived experience of suicide** |
| --- |
| **Affected by suicidality**   - How can I stay with the decision for life? What helps me? - What helps me to survive? / What helps me to deal with suicidal thoughts? - What happened before the suicide attempt? - From my perspective: Why does a person attempt suicide at a time in his/her life? /Why do people die by suicide? - How did I deal with the suicide attempt? What helps me? - How was the time after the suicide attempt for me? How did I feel? - What helped me in the time after the suicide attempt? - How can I deal with relapses? - What is the function of suicidality for me? - What experiences do I have with my own desire/ urge to suicide? - In which life situations did I think about it/was I close to taking my own life? How did I feel? - How did I manage not to suicide, how did I gain strength to continue living? - What does my deep crisis experience, the experience of feeling very close to death, mean to me? - What do I wish for myself and others in such deep crisis situations? - What is my current life situation? - How did it happen that I felt desperate/hopeless? - How is this desperation/hopelessness? What are these patterns of thought? - How do I get out of the despair or the thought patterns? How can I counteract this? - What would my advice be to people who are desperate? - From my perspective: What would be a good reaction if someone said that he/she is feeling bad, he/she is desperate/hopeless? / In my opinion, what could help other people with suicidal thoughts? /What do I want from others when talking about suicidality? - What was my experience when I went to a psychiatric clinic? - From my perspective: How to deal with a suicidal person? |
| **Loss of a close person by suicide**   - How was the time after the suicide for me? How did I feel? - What was my reaction to the suicide? - How did I deal with the suicide? - What helped me in the time after the suicide? - What would I have wished for from others in the time after my father's/mother’s death? - What does it mean for me to have lost my father/mother by suicide? - What do I want to give to others from my experience of suicide? / What would I like to share with other people affected and their relatives? |
| **Both**   - What does this experience mean for me? - How did I deal with stupid comments / incriminating reactions / burdensome reactions / stigmatization by the environment? What helped me? |
