# Supplementary material for: Development of an online suicide prevention program involving people with lived experience: ideas and challenges
Source: Res Involv Engagem. 2021 Sep 8;7:60. doi: 10.1186/s40900-021-00307-9 (PMC8424946; doi:10.1186/s40900-021-00307-9)
Supplement: Supplementary file 5 — Additional file 5. Content of the online suicide prevention program. [file 40900_2021_307_MOESM5_ESM.docx]

**Content of the online suicide prevention program**

Additional file 5. Content of the online suicide prevention program *8 lives – lived experience reports and facts on suicide* [„8 Leben – Erfahrungsberichte und Wissenswertes zum Thema Suizid“].

| Chapter 1: Demographics (“*About me*”) |
| --- |
| - Age, gender, level of education, size of residence - Own experience with suicidality or suicide (only one choice is possible that is currently most relevant to the person) |
| Chapter 2: Baseline assessment t_0_ (“*My thoughts*”) |
| - Literacy of Suicide Scale^1^ - Stigma of Suicide Scale: Self and Perceived ^2^ - Self-efficacy expectations of dealing with psychologically difficult situations^3^ - Distress^4^ |
| Chapter 3: Psychoeducation (“*My knowledge*”) |
| - Evidence-based health information: meaning of suicide or suicidality respectively, frequency of suicide, possible causes of suicidality, warning signs, precipitating events, risk and protective factors - Suicidality as a continuum - Excursus: Werther- and Papageno effect |
| Chapter 4: Experience reports on suicidality (“*My story*”) |
| - Video reports and text messages by persons with an experience of suicide: e.g. understanding suicide attempts or thoughts, helpful strategies to deal with suicidality from the perspective of affected persons (e.g. “What helped me to deal with suicidal thoughts?”; ”What helped me to deal with the suicide of a close person?”) - Suicide taboo: meaning and function of a taboo in general and for suicide in particular, reasons for tabooing suicide - Suicide stigma: meaning of stigma and stigmatization in general and concerning persons with experience of suicide, self-stigma, suicidality as consequence of stigmatization, difference between experienced and anticipated stigmatization, suicidality in various situations (migration background, serious physical diseases, higher age, homosexual or bisexual orientation, transgender) - Falsities concerning suicidality as opposed to reality - Possibility to anonymously communicate own experiences with suicidality or suicide |
| Chapter 5: Strategies I - Behavior, Mind, Body, Feelings (“*My coping*”) |
| Strategies to deal with or to prevent suicidality:   - Introduction of the concept of a safety plan in case of suicidality - Explanation of the link between behavior, body, thoughts, and feelings - Behavior: link between activity and well-being, creating a personal list of positive activities - Mind: cognitive restructuring technique, questioning thoughts - Body: Progressive muscle relaxation - Feelings: Psychoeducation about feelings |
| Chapter 6: Strategies II - Communication (“*Exchange*”) |
| - Strategies for communication with different groups of people (family, friends, doctors, psychotherapists) - Reflection on the disclosure of suicidality or suicide |
| Chapter 7: Personal goal setting (“*My goals*”) |
| - Personal goal setting according to “SMART” criteria (specific, measurable, agreed, realistic, and time specific) regarding to the strategies presented in Chapter 5 and 6. |
| Chapter 8: Post assessment t_1_ (“*Feedback*”) |
| - Literacy of Suicide Scale^1^ - Stigma of Suicide Scale: Self and Perceived ^2^ - Self-efficacy expectations of dealing with psychologically difficult situations^3^ - Distress^4^ - Feedback on the program (satisfaction, helpful elements) |
| “My space” |
| - Chapter navigation (successive unlocking of chapters; access to chapter 3-7 possible after completion of chapter 8)   “Library”   - Video experience reports in full length - Worksheets - Digital postcard messages from other program participants - References |
| Help options (“To find help”) |
| - Emergency numbers (e.g., crisis hotline) - Links to external help offers |
| ^1^Calear AL, Batterham PJ, Christensen H. The Literacy of Suicide Scale: Psychometric properties and correlates of suicide literacy. unpublished.  ^2^Batterham PJ, Calear AL, Christensen H. The Stigma of Suicide Scale. Psychometric properties and correlates of the stigma of suicide. Crisis. 2013;34(1):13-21.  ^3^New developed instrument  ^4^According to Mehnert A, Müller D, Lehmann C, Koch U. Die deutsche Version des NCCN Distress-Thermometers: empirische Prüfung eines Screeninginstruments zur Erfassung psychosozialer Belastung bei Krebspatienten. Zeitschrift für Psychiatrie, Psychologie und Psychotherapie. 2006;54(3):213-23. |

*Note: Chapters 3-7 each contain several short video sequences of eight people with a lived suicide experience who talk about their experiences, including what helped them in dealing with suicidality or the suicide of a close person. The focus of the interviews was, among other things, to convey hope, to show how to talk about the topic of suicidality and suicide, and to point out support options in crises.*
