# Supplementary material for: Development of an online suicide prevention program involving people with lived experience: ideas and challenges
Source: Res Involv Engagem. 2021 Sep 8;7:60. doi: 10.1186/s40900-021-00307-9 (PMC8424946; doi:10.1186/s40900-021-00307-9)
Supplement: Supplementary file 6 — Additional file 6. Reflection on the development process. 6.1 Evaluation of involvement: enabling and hindering factors in program development. 6.2 Lessons learned from program development. 6.3 Opinions on the lack of evaluation during program development. 6.4 Reflections on involving a lived suicide experience team in an online suicide prevention program. Development from researchers’ perspective. [file 40900_2021_307_MOESM6_ESM.docx]

**Reflections on the development process of an online suicide prevention program involving a lived experience team**

**From lived experience team’s perspective**

Summative evaluation of the involvement of the lived experience of suicide team in the development of an online suicide prevention program (N=7 lived experience team members)

Additional file 6.1 Evaluation of involvement: enabling and hindering factors in program development (N=7).

|  | Example(s) of a team member’s statement(s) |
| --- | --- |
| **Enabling factors in program development** | |
| **Overarching** **factors** in collaboration experienced as helpful | |
| Respect  Caring for each other  Acceptance of each other  Empathy  Getting to know each other better  Openness  Trust  Working at the same level  Transparency | “I felt accepted in the group. The atmosphere was very relaxed for the most part. We all treated each other very respectfully, I think. It was good for me to feel accepted in the group and to contribute to a valuable project and to get to know the others better.”  “Experiencing openness - honesty - understanding”  “I found the work within the team good, that we, first of all, had a good atmosphere, the trialogue took place, because we worked with each other at eye level. It was a project at eye level - not about, but with those who are affected.”  “We had a good discussion; it was positive that we argued about terms and were able to clarify them.”  “Sharing, hearing from others, sharing stories yourself; seeing that others feel the same way; realizing how many strategies you have; being treated respectfully + normally. It was before Corona! Just sitting together in a room. I found the interaction with each other (among those affected) nice, respectful, considerate.”  “Open atmosphere, trust, transparency, care, concern also for physical well-being”  “In this case, it was proven (once again?) that people with the same previous experiences can understand each other without many words. So-called outsiders can contribute greatly to a progressive process through restraint [e.g., by active listening]. Transparency promotes the willingness to open up. Appreciative work at eye level was always a given.” |
| Factors **within the team** experienced as helpful | |
| Good working atmosphere due to overarching factors. | *Please see above.* |
| Sufficient possibilities to bring own concerns into the project | “There was always enough time and opportunities to get involved.”  “Since I was able to express my needs, the involvement opportunity was just right for me.” |
| Sufficient space allows ideas to emerge within the group | “In the group discussion, we then get suggestions from the ideas and contributions of the others. If this happens very quickly, I take everything in, but usually can't react as quickly. However, I have seen that the others have stimulated each other in the process and that they have come up with more and more ideas. I think it's good to give space for everything.” |
| Consensus ability of the group | “It was possible to bring in dissenting opinions and we almost always managed to find a consensus.”  “Everything could be said and we then decided together whether or not to include it.” |
| Possibility of exchange outside of team meetings | “It's great to talk together, too, and exchange ideas when there's a need = uncomplicated + informal.”  “Discussions sometimes continue after meetings” |
| Team members stable enough for involvement | “Team members were burdened (some more, some less), but were all stable enough for involvement” |
| Factors of **team moderation / researchers** experienced as helpful: | |
| Good working atmosphere due to overarching factors | *Please see above.* |
| Sensitive, empathetic moderation/leadership of the group (MD & JB). | “Especially with the topic of suicidality, it could quickly become encroaching. It wasn't. It was empathetic.” |
| Safety feeling through possibility of contact with team moderation / project team (MD, JB, TB & SL) in potential (suicidal) crises, also beyond meetings through different communication channels (by email, phone or in person) | “Knowing that I can turn to [researchers] at any time when a problem arises, such as a heavy burden from dealing with my past experience with suicide, has greatly helped me feel in safe hands.”  “I also found the offer to talk to [researchers] if problems arise in connection with the work on the topic helpful. It made me feel well protected.” |
| **Organizational** **issues** experienced as helpful: | |
| Continuous information about team meetings and about the status of the project | “It was great that [researcher] called every time because I was not able to (read, write, etc.) e-mails.” |
| Sufficient time and opportunities to get involved; flexible extent of involvement | “Everyone could collaborate to the extent he/she wanted to.”  “At times I was a little bit left out, because I lived [not in Hamburg] for more than 8 months of the project development time, so I didn't participate in the discussion/design, but only worked on the content (text and video). But that was also my wish at the time, or I couldn't do otherwise.” |
| Importance of time between team meetings (possibility to prepare and follow up the team meetings) | “For me, it was nice to be able to send input/ideas via email even after the team meetings.”  “It was helpful that the materials to be reviewed were first sent to each person and then discussed in regular face-to-face meetings.”  “I can contribute best when I have some time to think about things in peace. So I found it particularly good when we were sent the questions we wanted to discuss in advance.” |
| Clear focus of team meetings; clear project framework | “The process of website/intervention development proceeded swiftly, at each meeting it was clear what it was going to be about and at the end we were always a bit further ahead.”  “Clear parameters had been defined, there were parameters that could be adjusted” |
| Selection of a suitable photographer/filmmaker who could sensitively deal with the topics of suicide and suicidality | “The filmer/photographer was really, really great. That is very important. He was sensitive, respectful, humanly open + honest, practiced + routine, patient (!) while waiting, clear, etc.,” |
| Financial reimbursement | “That the work of the team members was financially rewarded” |
| **Other issues** experienced as helpful: |  |
| Personal changes as a result of the project (evaluated positively) | “I took an even more nuanced look at suicide.”,  “Realizing how many strategies you have [to deal with suicidality]. It gave me courage, I became more aware of my own competencies and self/efficacy. That's a lot.”  “I have grown through the project.”  “On the whole, I would do it again exactly the same way. It was good that I had a therapist during the work on the project because sometimes the topic rumbled afterwards still in me. However, it also became clearer to me how I stand on the subject of suicide and what suicidal thoughts actually mean to me.”  “I have learned to listen patiently to opinions that differ from mine, which I was definitely less able to do before the project than I am now. I find this really helpful now, in associations, political work, profession and also to change my world view sometimes.” |
| Ruminating on issues after a team meeting | “Nothing was actually bad, except that sometimes it triggered something and then I lay awake at night thinking about it.”  When asked in the group about ruminating: “Ruminating wasn't all bad, it's also normal and also has something positive. Growing sometimes hurts a bit.”  “Through the project I got into motion internally, through the project framework there was the possibility to talk about it without having to explain oneself. Being a part of the team is a good way to cope.” |
| Project experienced as valuable | “It was enjoyable and I had the feeling throughout that I was working on something worthwhile.”  “It was good for me (...) to contribute to a valuable project.”  “I was proud that the project started well (...).” |
| Pluralism of experiences as enriching for the project | “What different perspectives different backgrounds of experience, biographies, etc. bring and how enriching these are for such a program.” |
| **Hindering factors in program development/challenges** | |
| Nothing in particular | “I don't think anything went badly at all. I can't think of anything that was less helpful either.” |
| Unpleasant feelings were activated in the team meetings when suggestions had been rejected | “In terms of procedures and approaches, suggestions and requests of mine that I actually found meaningful and constructive were often rejected. This made me feel passive/deprived of power and excluded.” |
| Overall project duration too long | “Overall too long and thus tiring. Technical implementation took far too long.” |
| Length of team meetings: Too little time in meetings | “In some parts, I think more time for discussion during the face-to-face meetings or more frequent face-to-face meetings would have been helpful/necessary, as there was often more to discuss than there was time.” |
| Number of team meetings: too few meetings - also too few non-project related meetings | “For once, I would have liked a meeting that was held explicitly for social reasons - not to work, but just to get to know each other. But maybe I just missed something like that when I wasn't in Hamburg.” |
| Wish for more personal contact between team members | “For such a profound topic, I would have expected more personal interaction [outside of the project].  “I would have liked to spend more time in the round, with more exchange. But some others wanted to leave right away (to other appointments or something).” |
| Formative evaluation did not take place | *Please see 6.3* |
| Continuation of the online program not discussed early enough | “In retrospect, I wish we had thought about and decided earlier how to proceed with the site [the online program "8 Lives"]. It would be ideal if it didn't have to be taken offline at all, i.e., if it simply continued to run without evaluation, in my opinion.”  “I was proud that the project started well and due to a misunderstanding, I was very disappointed that the site was closed and I am glad that it continues.”  “After the termination make clearer what has become of it. (Whereby by the Corona pandemic somehow also my energy was lost).”  ”Possibly clarify the extent to which the project can be continued permanently, funding/support for the future. But I also realize that at the beginning of a research project there are enough other problems in the foreground.” |
| Flow of information | “The calls were sometimes a little close beforehand. I didn't have the feeling of being informed at the same time as the others. Sometimes it was a bit of a rush for me or I didn't manage to answer the other day.” |

Additional file 6.2 Lessons learned from program development (N=7).

| **Category** | **Example(s) of a lived experience team member’s statement** |
| --- | --- |
| Continuation of the online program | “In retrospect, I wish we had thought about and decided earlier how to proceed with the site [the online program "8 Lives"]. It would be ideal if it didn't have to be taken offline at all, i.e., if it simply continued to run without evaluation, in my opinion.”  “I was proud that the project started well and due to a misunderstanding, I was very disappointed that the site was closed and I am glad that it continues.”  “After the termination make clearer what has become of it. (Whereby by the Corona pandemic somehow also my energy was lost).”  ”Possibly clarify the extent to which the project can be continued permanently, funding/support for the future. But I also realize that at the beginning of a research project there are enough other problems in the foreground.” |
| Greater involvement of persons affected | “Involve affected persons already in the development of the concept / application. I would have liked to be more involved in the planning of the evaluation (concept, selection of questionnaires, etc.)”  “The people we are talking about should have a say in the procedure as well, I think.” |
| Plan more financial and personnel resources | “Plan more financial and personnel resources for  -public relations/advertising for the program so that more people become aware of it.  - to make the program permanent/funded/supported in the future.  - for the evaluation of the program and the dissemination of its results.”  “Money for "catering" or that [researchers] don't have to pay for cookies etc. during some of the meetings.” |
| Interface between the technical and design online implementation and the project | “Persons who graphically and technically implement the intervention should be more/properly involved in the overall project (e.g. in the context of a job share, similar to research assistants, student assistants, or similar), so that they understand its concerns better and there is less loss of information and time loss at the interfaces”;  “Technical implementation took far too long.” |
| Plan a formative evaluation | *Please see 6.3* |
| Schedule additional meetings without a focus on the project | “I would have liked to have a meeting that is held explicitly for social reasons - i.e., not for work but just to get to know each other better.” |
| Explain more detailed specific tasks of project team members | “I found it exhausting that [project team members] seemed to be there - yet were never there. I found that incongruent. More transparency might have helped me - i.e., who does which tasks in the background.” |
| Improve flow of information for those without internet access | “The calls were sometimes a little close beforehand. I didn't have the feeling of being informed at the same time as the others. Sometimes it was a bit hectic for me or I didn't manage to answer the other day.” |
| Consider the individuality of a project and the people involved | “They [similar projects] should take our tips and then do their own thing.” |
| Additional psychotherapeutic support is important | “That psychotherapeutic expertise/counseling/support is important to professionally respond to conflicts with team members, crises, etc. of the participants.” |

Additional file 6.3 Opinions on the lack of evaluation during program development (N=7)*.

| **Category** | **Example(s) of a lived experience team member’s statement** |
| --- | --- |
| Sufficient opportunities to provide feedback | “There was always an opportunity for feedback on the program development process, which was also listened to and acted upon where possible”  “Due to the good personal contact my needs were met”  “[During team meetings] I didn't miss much - I said what was important to me. ” |
| **Advantages of a formative evaluation of the program development from the lived experience team members‘ view** | |
| Individual level | |
| Needs of members are perceived (in time); problems/difficulties noticed more quickly | “Is certainly useful and can help uncover potential problems/difficulties more quickly” |
| Regular feedback to perceive own needs | "We are encouraged to consciously pay attention to what we want, need, miss."  "We are given space and time to think about possible improvements, to pause." |
| Personal development/ self-reflection | "The process could be improved by this and the personal development of the individuals could be encouraged." |
| Shy people get a chance to speak up/ Expressing criticism can be difficult | “Criticism and feedback from shy people would be better heard.”  “It is not easy for most people to express criticism in a large group or to approach [the group coordinators] without being asked.” |
| Interest in feedback from others | “I am interested in the answers of others to these questions.” |
| Team level | |
| Team work even more improved by evaluation | "The collaboration in the group could be more intense, there could be more closeness if we learn more about each other, what our needs are." |
| Countermeasures possible | “(…) to counteract possible undesirable developments and to strengthen what is going well” |
| Appreciation of the work | “(…)it's also a kind of appreciation of our work” |
| Relief for group moderators/researchers | "Maybe [group moderators/researcher] would be relieved a bit, because then they wouldn't have to pay so much attention to how things are going in the group and how the individuals are doing, if this is regularly asked."  "Maybe it would be good for the people in charge [group moderators/researcher] to be asked and accompanied themselves." |
| Disadvantages of a formative evaluation of the program development from the lived experience team members‘ view | |
| Time factor | “The whole process would take longer.”  “It takes time.” |
| Interruptions of the (normal) workflow | “There would be interruptions, which could also be disturbing.” |
| Dislike of questionnaires | “I'm glad we didn't evaluate so much because I just have an aversion to questionnaires. The reason is that in psychiatry a lot is evaluated. The questionnaire partly replaces the conversation and because of some questionnaires some questionable diagnoses are attached. (...) I am especially against tick tests, because sometimes it is more important why I put the cross like that than the cross itself. I often put crosses because I more or less capitulated to the questions, because I would put the cross in different places depending on the situation.)” |
| Feedback sheet triggers pressure | “First of all, it reminds me of the feedback forms that you're always supposed to fill out quickly at the end of a training course and for which I actually need more time and therefore feel pressured every time. So spontaneously I have rather a negative feeling about it.” |
| Involvement/Team/Research in focus instead of topic of suicide/suicidality | |
| Distraction from actual topic and focus on evaluation/team / Desire to focus on people, their issues and not on research | “I might already be thinking about the evaluation questions during the group discussions and would thus be distracted from the actual topic.” |
| Evaluation of teamwork as annoying | “It could also be annoying to have to constantly evaluate the collaboration.” |
| Being and working together becomes artificial | “The process becomes more scientific (I'm not quite sure that's the right word), possibly at the expense of the natural, self-evident development in being and working together.” |
| Circling around teamwork | “Perhaps we get too bogged down in conversations that revolve around collaboration.” |
| Pseudo feedback / Feeling like a guinea pig | “No, I would have felt more like a "guinea pig" due to standardized formalities. This way I always had the feeling that it was about me and not about the research.” |
| Ideas for designing a formative evaluation | |
| Written vs. oral questioning | “I would have liked to do it in writing and then discuss it in the group, that is, say it myself and not hand it in without comment.”  “I think verbally is better, leads to fewer misunderstandings, to write down interpersonal is difficult. But written would also work.” |
| Frequency of evaluation | “Once after half [of the program development] and once at the end [of the study] like now. Then you can change something and you're not interrupted too often in the flow of work. In the end, I think it's good to think about it again, it's also a kind of appreciation of our work.”  “Depends on the length of the project and the frequency of interactions; in any case once at the beginning (for expectations etc.), once in the middle (to counteract possible undesirable developments and to strengthen what is going well, see above) and at the end (see above).  Depending on the intensity of the collaboration, an additional process evaluation 1-2 times a year would be good - alternatively, something like this can also be built into the regular meetings on a small scale.”  “Maybe it would be good to do such an evaluation about halfway through and again like now at the end. In between again and again would probably be too much for me.” |
| Feedback of formative evaluation | “During program development, I think it makes sense to ask questions so that the results can still be incorporated and improve the process; here I would find it useful to ask questions alone and in a group: In the group to discuss things that the others are also concerned about and can be discussed in a larger group; alone to discuss things that do not concern the others and/or cannot be discussed in a larger round. After the end of the program development, I find a summative survey useful to be able to draw lessons from the completed overall process for next processes (and to be able to communicate these to others).”  “I would then like to have the results discussed in the group after the evaluation. If we decide to do this, it should also be communicated to the others so that everyone knows clearly what the status is and so that we can change something if it is desired and it seems to make sense to the group.” |
| Discuss purpose for formative evaluation transparently | “That the people involved would be made to understand [/would receive an explanation] what this evaluation is being conducted for and why it is useful. Is it to improve collaboration, is it to be studied scientifically, or is it a place for content criticism?” |
| Questions on formative evaluation that the lived experience team would find appropriate | |
| Suggestion 1 lived experience team member | - What are your goals, what do you want to achieve in the involvement/collaboration/work? - What do you expect from others and from yourself? - What do you need to achieve your goals, from the group, from [group moderation/researchers], from yourself? - How do you perceive the involvement/collaboration/work, the process, the interaction with each other? - How does it feel to work on the topic of suicidality/suicide? - How does the involvement/collaboration/work feel? Do you want something to be changed? |
| Suggestion 2 lived experience team member | - What would make our project better? - Do you feel comfortable in the group? - What do you need to feel more comfortable in the group? - What would you like to know/didn't you understand about the project? - A free text field |
| Questions from this evaluation sheet | *Evaluation of the involvement*   - From your point of view, what is going well in the involvement/collaboration within the team to develop the "8 Lives" program? What was helpful? - From your point of view, what is going badly in the cooperation within the team for the development of the program "8 Lives"? What was less helpful or not helpful at all? - Are your concerns sufficiently taken into account in the cooperation within the team? Were you able to contribute sufficiently? - What are your other experiences with the development of the program "8 Lives" regarding the work/involvement within the team?   *Lessons learned*   - What would you recommend to another group if they wanted to develop an online program on suicidality / antistigma-program? What would you do differently if we could start over with the development of "8 Lives"? - Would you like more or less involvement at some points? If yes, please specify when/how? (if applicable, why?) - Do you need anything at some points during the development? If yes, please specify what and when? (if applicable, why?) - Other lessons you learned from the development process: |

*In the post-evaluation of the summative evaluation, the lived experience team suggests for other PPI projects to first discuss in detail the purpose of the formative evaluation and its advantages and disadvantages, to decide together whether a formative evaluation should take place or not. If the team decides to do a formative evaluation, the questions, frequency, type of feedback etc. should be decided together as a team.

**From researchers’ perspectives**

Evaluation of the involvement of the lived experience of suicide team in the development of an online suicide prevention program (N=3 researchers)

Additional file 6.4 Reflections on involving a lived experience of suicide team in an online suicide prevention program development from researchers’ perspective (N=3)

| From the perspective of the researchers, the following aspects were helpful in working together to develop an online suicide prevention program:  **Enabling factors / Ideas** | |
| --- | --- |
| **Working** **atmosphere** | “Working atmosphere of respect and openness to the experiences of others and in which one can feel comfortable. We also found it important to pay attention to little things, e.g., enough space, water, snacks etc.”  “When developing the online program, we called the team "working group" (not “lived experience team”). Of course, lived experience plays a role, but working together on the program was the focus.” |
| **Trust via dual role** | “One person with a scientific background in mental health care research has also a lived experience of suicide. This might have made it easier for other persons with a lived experience to share these.” |
| **Good group cohesion** | “By the participation in the association Irre menschlich Hamburg e.V. persons knew and trusted each other and were used to talk about lived experiences. This increased openness and quickly led to good group cohesion.” |
| **Autonomy** | “Giving as much autonomy and decision-making options as possible (e.g. different ways to participate, own decisions (also different from the plan) were emphasized). Each team member could decide for themselves how much space the subject of their own lived experience would have in the project.” |
| **Availability of the project team/giving emotional support** | “Dealing with suicidality and suicide is very emotional. Making it clear to the group that they had the opportunity for questions and exchange with different  project team members through various channels (telephone, mail, meetings, post).  At the beginning and end of a meeting, time was scheduled to talk about own feelings (also regarding the project). Sensitivity for feelings in the group and direct addressing was helpful because the topic of suicidality and suicide is 1. emotionally demanding and 2. stigmatized.” |
| **Transparency and continuous contact** | “Clear information to all team members about the project status (e.g. technical delays in the implementation of the online program; protocol summary to everyone to keep everyone up to date and involved, continuous feedback on the progress).” |
| **Valuing opinions/ideas and joint consensus** | “We took opinions and ideas seriously, even if they were, for example, ideas that must be clearly rejected from a scientific perspective. Some topics were discussed controversially. The project team made final decisions on critical issues. We presented the reasons for a decision in a transparent manner as well as value ideas from the lived experience team and, wherever possible, found a compromise.” |
| **Information on program involvement for therapists** | “If a lived experience team member has been in psychological treatment, we have recommended that they should mention their involvement in program development. It is helpful having the option talking to a therapist who is independent of the project team. The decision to talk to the therapist about involvement was left up to each member. There was also the possibility for therapists to contact the project team in case of questions.” |
| **Structure** | “Clear structure of the meetings (who moderates, agenda, clear task distribution and deadlines). Between the meetings there was enough time for reflection. All meetings had a rough time schedule but could be held longer or shorter if necessary.” |
| **Feedback** | “Feedback given on the incorporation of the text reviews.” |
| **No peer pressure** | “Making sure that no peer pressure, e.g. in disclosure, is created. We communicated that it is important that everyone is sensitive to her/himself. This was supported by the possibility to exchange with a person from the project team outside of the team meetings.” |
| **Clarification of possible risks** **of participation** | “Clarification of possible risks of participation in advance (e.g. that it is not possible to prevent material from being downloaded and distributed by unauthorized persons). Due to the additional rooting in the established association Irre Menschlich e.V. it could be ensured that the participants were stable enough to deal with the program development.” |
| **Allowance** | “An allowance rewards the work of the team and expresses appreciation for the courage and engagement. It was ensured that a video report on lived experience is not made solely for financial reasons. The possible consequences of a public disclosure were discussed and reflected upon in advance. It was possible to withdraw the video at any time (also before it was published) without this having any effect, e.g. on the expense allowance.” |
| **Common message** | “It was important to find a common message to send regarding suicidality conveying hope and encouraged people to continue living.” |
| The project team experienced the following challenges in working together to develop an online suicide prevention program:  **Hindering factors / Challenges** | |
| **Reflection on own role** | “Important to reflect to take on the role in participatory exchange and not to act in the role of psychologist or therapist. It is also important for researchers to reflect their own attitudes and feelings towards suicidality and suicide.” |
| **Emotionally challenging** | “Empathizing with people who share their lived experience of suicide is more emotionally challenging than dealing with suicide "on paper". From the project team's point of view, group discussions and conducting the interviews was emotionally challenging, as was the subsequent checking of the edited video sequences. Supervision and intervision were helpful. Talking about the lived experience of suicide in front of a camera is emotionally taxing. Since suicidality and suicide is an emotionally loaded, challenging and stigmatized issue, researchers should reflect on their own attitudes on suicide and suicidal persons and feelings. It might be interesting to study transference and countertransference phenomena in the context of suicide research (both of researchers and team members), which was not considered in our work.” |
| **Distinction of non-discriminatory behavior and the assessment of functional impairment** | “The intention to behave in a non-discriminatory manner must be distinguished from the assessment of an actual functional impairment. We experienced that it can be a fine line to comply with a duty of care for persons who are at higher risk of suicidal behavior and at the same time not to discriminate unintentionally.” |
| **Dealing with a possible (suicidal) crises of a team member** | “Even if the persons consider themselves as stable enough for the involvement, it must be defined how to proceed in (an unlikely) case of a crisis or an emergency case in advance with the lived experience team and project team - without taking over the role of a health care professional. In retrospect, we considered to define the procedure voluntarily for all project participants, regardless of the lived experience of suicide. It must be determined who is the contact person in a possible crisis (preferably a health care professional independent of the project). It might be useful to collect telephone numbers of a close person of the team members who can be contacted in an emergency case. It was agreed with the team members that in case suicidality increases, in addition to the health care professional the project team should be informed about it.  *Increase of suicidality/deterioration of condition:* We discussed the possibility of a deterioration of a team member’s condition at the first meeting. In our team we decided together that it is not expedient to plan in advance for every possibility that might arise in the course of the project, how we can then continue and shape our work. We decided to discuss on factual situations at the given time. One person could not attend one lived experience team meeting due to an inpatient clinic stay (because of an increase in suicidality). We discussed with the person and the team how we should deal with the specific situation and have decided for a team meeting without the person. However, retrospectively, we would discuss how to deal with a possible inpatient clinic stay of a team member at the beginning when a lived experience team is formed because non-attendance due to an inpatient stay can have an impact on the group.” |
| **Dealing with ambiguous messages regarding suicide** | “Making clear in advance that potentially ambiguous or ironic messages regarding suicide should not be sent by e-mail, as the seriousness of the message cannot be assessed by the project team and will result in the message being treated as an emergency (which happened one time during program development).” |
| **Group domination** | “As in a lot of groups, there are members who dominate the discussion more than others. For the moderation it is important to maintain a balance. It helped to actively involve other team members in their views on the initial question. It is also helpful to lead the moderation in two persons.” |
| **Focus/Thematic excesses** | “Certain topics can be debauched in the context of program development (e.g., own suicide experiences described multiple times). In our opinion, there should be room for this in lived experience team meetings, but it should be limited if the discussion is too extensive or if it becomes too exhausting for other members. In the meetings, there was an opportunity to suggest a break or to leave the room if one needed a break.” |
| **More breaks in a meeting** | “In retrospect, more fixed breaks in a meeting could have been planned.” |
| **Group dynamics** | “During the meetings it could be challenging to bring persons with different lived experiences of suicide together, e.g. dynamics can arise between members. We would recommend setting up a team in which people with different suicide experiences are evenly distributed which was not given in our team. Depending on the goal of a collaboration, an idea would be to put teams together as homogeneously as possible in their experiences.” |
| **Ongoing involvement / Continuation of the program** | “The continuity of the program should have been discussed earlier in the team.” |
| **Link between the technical implementation and the team** | “The technical implementation of the online program took a very long time (thus postponing team meetings). A closer link between the technical implementation and the team could have been helpful.” |
